# Dr. Ralph Lyman Parsons

**Published:** 1914-04

**Authors:** 


Dr. Ralph Lyman Parsons, N. Y. Medical College, 1857, died at his home in Ossining, N. Y., February 26. He was born in Prattsburg, Steuben County, N. Y., July 30, 1828, and was educated at Amherst, graduating in 1853. He was prominent in neurology, having been superintendent of the New York City Asylum for the Insane from 1865 to 1877; of the Kings County Asylum from 1877 to 1878, and established a private hospital Ossining in 1880, of which he remained in charge till his death.

